# Schwannoma of the submandibular gland in neurofibromatosis 1: a case report

**DOI:** 10.1093/jscr/rjaf043

**Published:** 2025-02-28

**Authors:** Hephzibah Grace Mandam, Swathi Santharaj, Noor Ul Hassan

**Affiliations:** Shri Atal Bihari Vajpayee Medical College and Research Institute, Lady Curzon Rd, Shivajinagar, Bangalore 560 001, India; Shri Atal Bihari Vajpayee Medical College and Research Institute, Lady Curzon Rd, Shivajinagar, Bangalore 560 001, India; Bowring and Lady Curzon Hospital, Bangalore 560 001, India

**Keywords:** submandibular schwannoma, schwannoma, neurofibromatosis 1, excision, submandibular gland, case report

## Abstract

Schwannoma is a well encapsulated benign nerve sheath tumor of neuroectodermal origin with an indolent progression unless associated with malignancy. Schwannomas arise from the Schwann cells as do neurofibromas and are usually solitary except in Von Recklinghausen’s disease. The occurrence of a schwannoma in the head and neck region is about 25%; however, submandibular gland schwannoma is a rare form of an extracranial neurogenic tumor arising from peripheral, central, or autonomic nerves. We report a rare case of a submandibular schwannoma in a patient with neurofibromatosis 1 which presented as a painless swelling in the submandibular region, treated by total excision of the submandibular gland. There was no postoperative deficit or recurrence within 3 months of follow-up. Our findings in this case suggest schwannoma of the salivary gland as a differential diagnosis of swelling in the submandibular area although rare, especially in a patient with neurofibromatosis 1.

## Introduction

Salivary gland schwannoma is an extremely rare form of extracranial neurogenic tumor. It is a slow growing, benign, encapsulated tumor of the Schwann cells. These tumors occur regardless of age or sex and are painless, insidious, and slow growing, rarely showing a rapid course [1]. Neurofibromatosis type 1 (NF1) is an autosomal dominant neurocutaneous syndrome that causes multiple central and peripheral nerve sheath tumors [2]. Here, we present a case of a schwannoma of the submandibular gland in a 40-year-old female patient. Our findings in this case indicate good prognosis with total excision of the submandibular gland. In this case, no neurological deficits or recurrence were noted after 3 months of follow-up.

## Case report

A 40-year-old female patient of Indian ethnicity presented to our surgical outpatient department with complaints of a painless swelling in the right submandibular region for 3 years. The swelling was insidious in onset and gradually progressive over the last 3 months. She is a known case of neurofibromatosis 1. Her medical history was otherwise insignificant.

Physical examination disclosed a well-nourished, moderately built woman with normal vital signs. On examination, a solitary, non-tender, smooth surface, firm, freely mobile swelling with well-defined borders, 3 × 3 cm in its largest diameter was palpable in the right submandibular region. No thrill/bruit was heard on auscultation. No regional lymphadenopathy detected. Oral cavity and ENT examination were normal.

**Figure 1:**
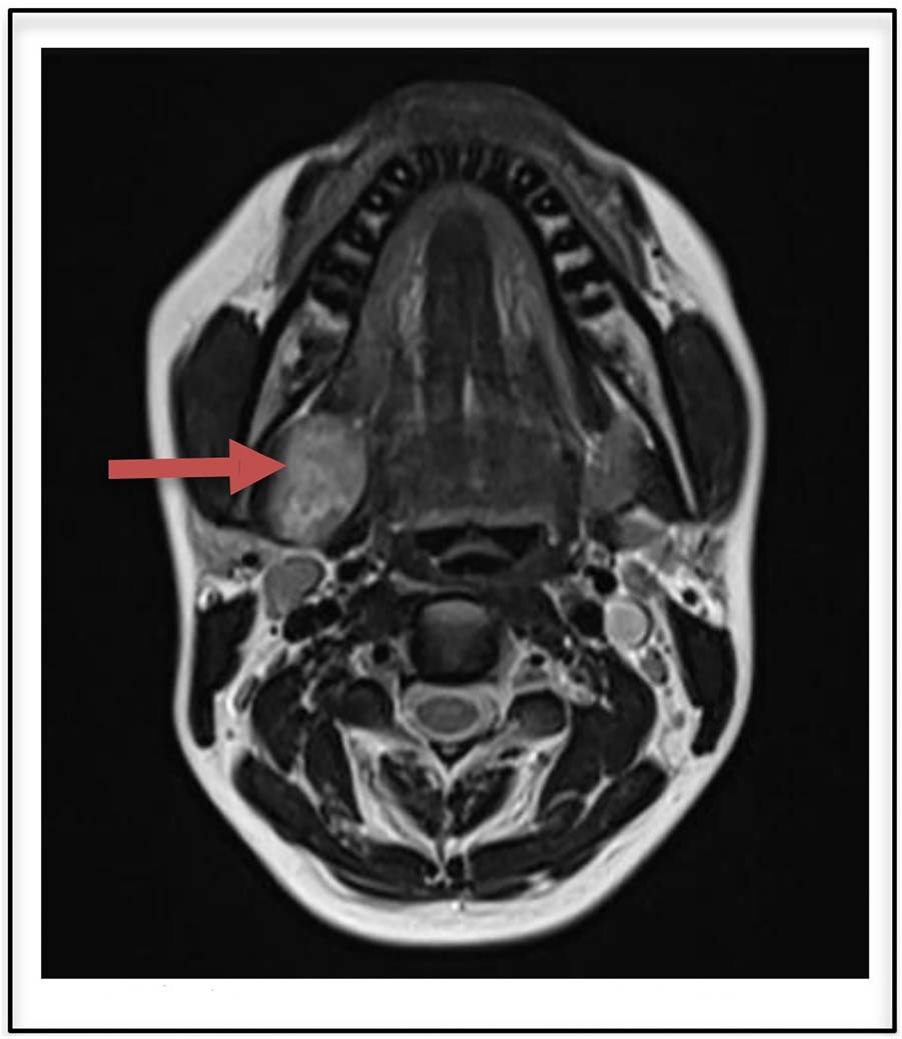
T2 heterogenously hyperintense oval shaped well circumscribed lesion measuring 2.9 × 2.7 cm on MRI (arrow).

Routine laboratory investigations were normal. Ultrasound scan of the neck region showed a hypoechoic lesion 2.9 × 2.7 cm in the right submandibular gland taking internal vascularity without calcification or necrosis. MRI imaging revealed a T2 heterogeneously hyperintense lesion with internal areas of low signal areas giving rise to a fascicular pattern that is typical of nerve sheath tumors (Fig. 1). On correlation with ultrasound, the lesion appears heterogeneously hypogenic with moderate internal vascularity.

A fine needle aspiration cytology of the mass showed spindle shaped cells without atypia which was inconclusive.

Our patient was then prepared for surgical evaluation and resection. Total excision of the right submandibular gland was done under general anesthesia. The mass was carefully dissected from its adjacent structures. The lesion was completely excised with the submandibular gland and the surgical wound was closed.

**Figure 2:**
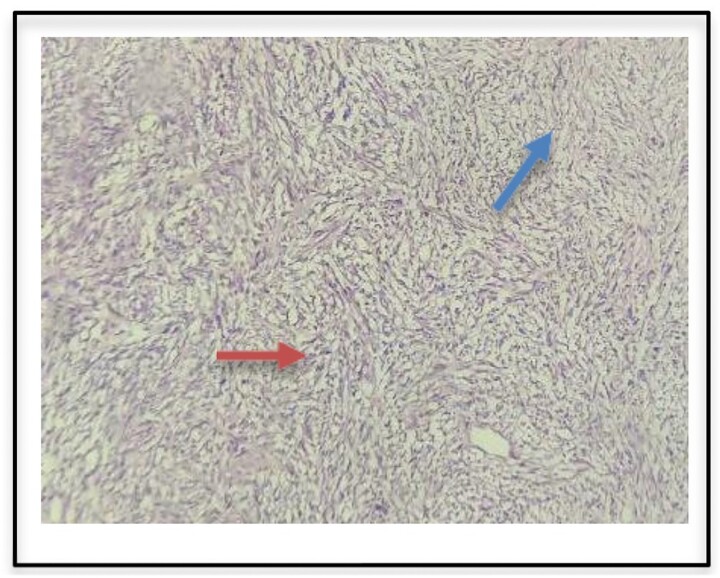
Histopathologic examination of surgical specimen indicates Antoni A (red arrow) and Antoni B bodies (blue arrow).

Histopathological examination as seen in Fig. 2 confirmed seromucinous salivary gland tissue along with hypertrophic neurovascular bundle. Adjacent areas showed well circumscribed tumor tissue composed of hypercellular (Antoni A) and hypocellular (Antoni B) areas of proliferating spindle cells with wavy serpentine nuclei and pointed ends. Occasional foci showed palisading of nuclei (Verocay body). These findings were suggestive of a schwannoma.

**Figure 3:**
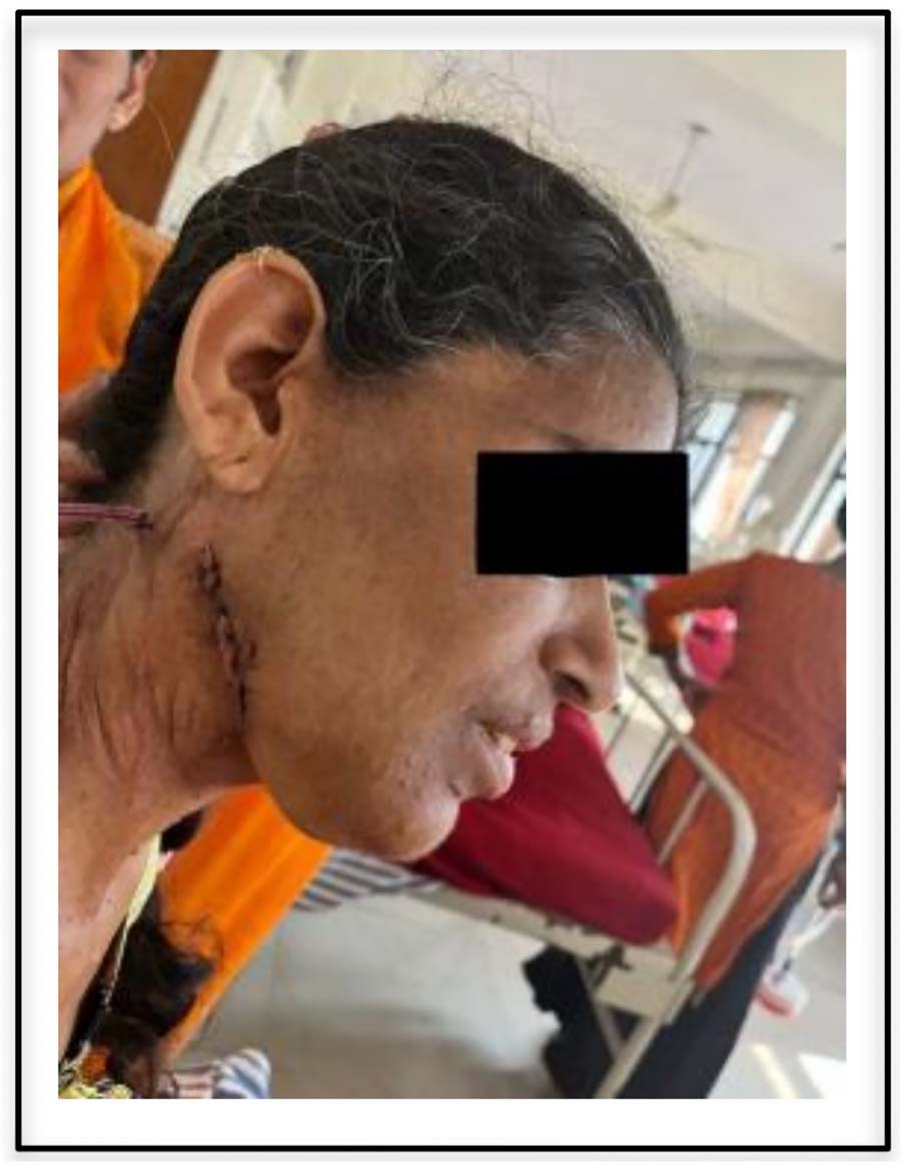
Postoperative view of incision site.

The patient had an uneventful postoperative recovery and no neurologic deficits or recurrence was noted within 3 months of follow-up (postoperative incision shown in Fig. 3).

## Discussion

Schwannomas are solely derived from Schwann cells and are usually solitary lesions except in Von Recklinghausen’s disease where multiple neurilemmomas are seen [3]. About 25–45% of schwannomas occur in the head and neck region [4]. While the nerve of origin may be either peripheral, central, or autonomic in 10–40% of cases, the nerve of origin is unidentified [5]. Schwannomas are slow growing tumors and usually present between the second to fourth decades of life [6].

Schwannoma of the salivary gland is a rare form of an extracranial neurogenic tumor, with most presenting in the parotid gland originating from a peripheral branch of the facial nerve. Other nerves of origin in the head and neck are the sensory divisions of cranial nerves, most commonly the vestibular nerve and the vagal nerve [7]. Neurilemmomas of the peripheral portion of cranial nerves usually affect soft tissues of head and neck with the most common site being the tongue, while the lower oral cavity is an unusual site for these tumors [8].

Clinically, the tumor does not present for a long time. The most common complaint is a slow growing mass. Neurological symptoms and pain are rare. In our case, the patient did not complain of pain, odynophagia, or dysphagia. Malignant transformation of solitary schwannomas is usually rare; however in neurofibromatosis 1, there is a 10% chance of developing malignant peripheral sheath tumors (MPNSTs) [2].

Plain radiographs are not useful in establishing the diagnosis of schwannomas. Diagnostic investigations include computed tomography (CT), magnetic resonance imaging (MRI), and ultrasound scan. Ultrasound reveals a well circumscribes mass which is heterogeneous. On CT, schwannomas demonstrate low to intermediate attenuation on enhanced CT and variable enhancement on contrast enhanced CT. MRI is the best choice in detecting the extent of the tumor and correlates well with operative findings.

The MRI appearance of our case is identical to those reported in the previous literature showing low to intermediate signal intensity on nonenhanced T1-W1 and strong enhancement without enhancing cystic spaces on contrast-enhanced T1-W1 [9]. The heterogeneous, mottled appearance on contrast MRI with the presence of intratumoral vessels and the delineation of the tumoral capsule from the displaced submandibular gland strongly suggested a schwannoma. Other submandibular gland lesions included submandibular lymphadenitis and cervical cyst. The origin of the tumor a nerve can be indicated during imaging as split-fat, fascicular, and target signs [10]. These findings however could not be appreciated in our case.

The diagnosis of a schwannoma is confirmed histopathologically by the presence of Antoni A (hypercellular) and Antoni B (hypocellular) cells.

Previous literature reports that schwannomas tend to have cystic change due to hemorrhagic necrosis accompanying the increase of the tumor [10]. No evidence of cystic change or necrotic degeneration was seen. Treatment of schwannomas is surgical excision with preservation of nerve function due to resistance to radiotherapy. Recurrence is uncommon [11]. Often surgically treated cases exhibit postoperative neural deficits due to iatrogenic injury either to the nerve of origin or adjacent neural ending. In our case, no neural deficit was noted postoperatively.
